# Effect of an low-energy Nd: YAG laser on periodontal ligament stem cell homing through the SDF-1/CXCR4 signaling pathway

**DOI:** 10.1186/s12903-023-03132-6

**Published:** 2023-07-19

**Authors:** Nan Wu, Jianing Song, Xin Liu, Xiangtao Ma, Xiaoman Guo, Taohong Liu, Mingxuan Wu

**Affiliations:** 1 Hebei Key Laboratory of Stomatology, Department of Periodontology (II), Hebei Clinical Research Center for Oral Diseases, School and Hospital of Stomatology, Hebei Medical University, Zhongshan East Road 383, Shijiazhuang, 050017 Hebei People’s Republic of China; 2 Hebei Key Laboratory of Stomatology, Department of Laser Medicine, Hebei Clinical Research Center for Oral Diseases, School and Hospital of Stomatology, Hebei Medical University, Shijiazhuang, 050017 Hebei People’s Republic of China

**Keywords:** Low level laser therapy, Periodontal ligament stem cells, Endogenous periodontal regeneration, SDF-1, CXCR4 signaling pathway

## Abstract

**Background:**

The key to the success of endogenous regeneration is to improve the homing rate of stem cells, and low-energy laser is an effective auxiliary means to promote cell migration and proliferation. The purpose of this study was to observe whether low-energy neodymium (Nd: YAG) laser with appropriate parameters can affect the proliferation and migration of periodontal ligament stem cells (PDLSCs) through SDF-1/CXCR4 pathway.

**Methods:**

h PDLSCs were cultured and identified. CCK8 assay was used to detect the proliferation of h PDLSCs after different power (0, 0.25, 0.5, 1, and 1.5 W) Nd: YAG laser (MSP, 10 Hz, 30 s, 300 μ m) irradiation at 2th, 3rd,5th, and 7th days, and the optimal laser irradiation parameters were selected for subsequent experiments. Then, the cells were categorized into five groups: control group (C), SDF-1 group (S), AMD3100 group (A), Nd: YAG laser irradiation group (N), and Nd: YAG laser irradiation + AMD3100 group (N + A). the migration of h PDLSCs was observed using Transwell, and the SDF-1 expression was evaluated using ELISA andRT-PCR. The SPSS Statistics 21.0 software was used for statistical analysis.

**Results:**

The fibroblasts cultured were identified as h PDLSCs. Compared with the C, when the power was 1 W, the proliferation rate of h PDLSCs was accelerated (*P* < 0.05). When the power was 1.5 W, the proliferation rate decreased (*P* < 0.05). When the power was 0.25 and 0.5 W, no statistically significant difference in the proliferation rate was observed (*P* > 0.05). The number of cell perforations values as follows: C (956.5 ± 51.74), A (981.5 ± 21.15), S (1253 ± 87.21), N (1336 ± 48.54), and N + A (1044 ± 22.13), that increased significantly in group N (*P* < 0.05), but decreased in group N + A (*P* < 0.05). The level of SDF-1 and the expression level of SDF-1 mRNA in groups N and N + A was higher than that in group C (*P* < 0.05) but lower than that in group A (*P* < 0.05).

**Conclusions:**

Nd: YAG laser irradiation with appropriate parameters provides a new method for endogenous regeneration of periodontal tissue. SDF-1/CXCR4 signaling pathway may be the mechanism of LLLT promoting periodontal regeneration.

## Introduction

Periodontitis is the main cause of adult tooth loss. The fourth national epidemiological survey showed that 86% of the patients had periodontitis in the China [1]. The ultimate goal of periodontitis treatment is to regenerate damaged or even missing periodontal tissue. At present, the treatment effect of periodontal regenerative therapy is poor and has certain limitations [2], and the clinical transformation efficiency of periodontal tissue engineering research is low for various reasons. Since 2010, Kim et al. [3] successfully achieved periodontal tissue regeneration in animals without ex vivo cell transplantation. Tissue regeneration research centering on stem cell homing, such as endogenous regeneration, has gradually become a new hotspot in the field of regenerative medicine [4, 5]. Mesenchymal stem cells are the seed cells of endogenous regeneration. Successfully homing to the periodontal microenvironment and improving the homing efficiency during ischemia, hypoxia, and injury are issues that must be resolved before endogenous regeneration is used as a reliable and effective way of treatment in patients.

Basic and clinical studies have found that low-level laser therapy (LLLT) can transfer energy to cells and tissues through biological stimulation, promote cell recruitment and migration, and guide biological tissues to exert regenerative potential [6–8]. Studies have confirmed that pretreatment with hypoxia, drugs, exosomes, and so forth, can promote the recruitment and migration of stem cells or progenitor cells in the body to the injured site, and is related to the SDF-1/CXC4 signaling pathway [9, 10]. However, studies on whether the biological stimulation effect of low-energy lasers can change the periodontal microenvironment through this pathway to improve the homing rate of h PDLSCs are few. For LLLT, the factors such as wavelength, power, energy density, and irradiation time affect cell proliferation and migration.

Therefore, in this study, low-energy Nd: YAG with different powers was used to irradiate h PDLSCs, and the effects of low-energy Nd: YAG on the proliferation and migration of h PDLSCs were observed. The changes in SDF-1 expression were evaluated at the protein and gene levels to explore whether the mechanism of low-energy Nd: YAG affecting the proliferation and migration of h PDLSCs was related to the SDF-1/CXCR4 pathway. It provided a new means to improve the homing rate of mesenchymal stem cells. The selection of appropriate low-energy laser parameters also provided a reference for clinical application.

## Materials and methods

### Cell culture

Premolars or third molars that needed to be extracted for orthodontic treatment in patients aged 13–20 years were selected. The one third of the root periodontal ligament tissue was scraped, cut into about sections measuring 1 × 1 × 1 mm^3^, and placed in a culture flask. Then, 2 mL of complete medium was added, followed by incubation in a carbon dioxide incubator at 37℃. The flask was turned after 4 h. The medium was changed every 3 days, and the cells were sub-cultured when they grew to 80%–90% confluence, inoculated, and passaged at a ratio of 1:3.

## Cell identification

### Osteogenic induction and Alizarin red staining

The second-generation h PDLSCs were seeded in six-well plates at a cell density of 2 × 10^4^/mL, When the cells grew to about 70%–80% confluence, the osteogenic induction medium was added and the cells were placed in the incubator in the dark. The culture was induced for 21 days, and the cells were fixed with 4% PFA for 30 min and stained with 2% Alizarin red for 30 min. The formation of mineralized nodules was observed and photographed under an inverted microscope (Olympus Corporation, Japan).

### Adipogenic induction and oil red O staining

The second-generation h PDLSCs were seeded in a six-well plate at a cell density of 2 × 10^4^/mL, When the cells grew to about 100% confluence, the adipogenic induction medium was added and the cells were cultured for 21 days in the incubator. The cells were fixed with 4% PFA for 30 min and stained with saturated oil red O for 10 min. The lipid droplet formation was observed and photographed under the inverted microscope.

### STRO-1 immunofluorescence

Three slides were placed in 24-well plates, and 2 × 10^5^ cells/well were added. Cell-attached coverslip and then were fixed with 4% PFA, washed with phosphate-buffered saline (PBS) for 5 min thrice, and placed at 4℃ overnight. On the waterproof film, a glass slide was kept on 50-µL membrane-breaking liquid for 2 h, and then 50 µL of the primary and secondary antibodies were added. The cells were incubated for 2 h and stained with DAPI for 5 min. One drop of Fluoromount-G was added to each slide, and the slide was covered with cells. The cells were observed and photographed under an immunofluorescence microscope(Olympus Corporation, Japan).

### Flow cytometry

The second-generation h PDLSCs were digested and counted. The liquid containing 10^6^ cells was taken and centrifuged at 1100 rpm for 5 min. The cells were washed twice with 200–400 μ L of PBS and resuspended in 100 μ L of PBS solution. Then, 2 μ L of CD45PE, CD34APC, and CD44APC antibodies were added to each test tube in the dark, and the cells were incubated for 40 min and examined by flow cytometer(GE, USA).

### Experimental design

Different powers (0, 0.25, 0.5, 1, and 1.5 W) of Nd: YAG irradiation (MSP, 10 Hz, 30 s, 300 μ m) were used to irradiate h PDLSCs. The proliferation of h PDLSCs was detected by the CCK-8 method on the first, second, third, fifth, and seventh days, and the best irradiation parameters were selected. Then, the cells were divided into five groups: control group (C), SDF-1 group (S), AMD3100 group (A), Nd: YAG laser irradiation group (N), and Nd: YAG laser irradiation + AMD3100 group (N + A). Transwell assay was used to observe the migration of h PDLSCs. ELISA and RT-PCR were used to evaluate the SDF-1 expression after Nd: YAG laser irradiation.

C group: h PDLSCs were seeded in the upper chamber of Transwell, without a lower chamber.

S group: h PDLSCs were seeded in the upper chamber of Transwell, and SDF-1 was added to the lower chamber.

A group: h PDLSCs + AMD3100 were seeded in the upper chamber of Transwell.

N group: h PDLSCs cells + Nd: YAG laser stimulation were seeded in the upper chamber of Transwell, without a lower chamber.

N + A group: h PDLSCs cells + Nd: YAG stimulation + AMD3100 were inoculated in the upper chamber of Transwell, without a lower chamber.

### Laser irradiation method

The periodontal ligament stem cells were digested and 1 ml cell fluid was added to a 1.5 ml cylindrical centrifuge tube. The centrifuge tube is wrapped with alayer of tin foil to prevent light transmission, and then marked at a distance of 1 cm from the liquid surface of the centrifuge tube. The next, the cells wereirradiated with a 300 µm fiber of a neodymium laser using a ' z ' shape at the marked point.

### Cell proliferation assay

Nd: YAG laser (MSP, 10 Hz, 1 cm, 300 μ m) was used to irradiate the resuspended h PDLSCs for 30 s at powers of 0.25(9.54 J/cm^2^)W, 0.5(19.11 J/cm^2^)W, 1(38.22 J/cm^2^)W, and 1.5(57.32 J/cm^2^)W, and then the cells were inoculated in 96-well plates with 5000/well h PDLSCs. About 100 µL of the cell suspension was taken per well, and four duplicate wells were set and cultured in an incubator at 37 °C for 24 h. On the 1st, 2nd, 3rd, 5th, and 7th days, 10 µL of CCK-8 was added to each well and cultured for 30 min. The optical density (OD) at 450 nm was measured using a spectrophotometer(Bio-Tek USA).

### Transwell test

h PDLSCs were starved for 24 h, digested, irradiated with an Nd: YAG laser (MSP, 10 Hz, 1 W, 30 s, 300 μ m), and resuspended in 100 μ L of serum-free medium. The 10,000/cm^2^ cells were inoculated in the upper chamber. The medium containing 5% FBS was added to the lower chamber. Then, 100 ng/mL SDF-1 factor was added in group S, and 5 μg/mL AMD3100 factor was added in groups A and N + A.

### ELISA and quantitative RT-PCR

The h PDLSCs were seeded in six-well plates in groups C, A, N, and N + A. After 24 h, the cell supernatant was collected and detected using an SDF-1 ELISA detection kit (Sangon Biotech Co. Ltd). Then, 1 mL of TRIzol reagent was added to each well, and the lysate was transferred into a 1.5-mL centrifuge tube. Total RNA was extracted and reverse transcribed into cDNA following the manufacturer’s protocols for the TRIzol kit. Associated gene primers were designed based on RT-PCR. The synthesized primer sequences are shown in Table 1. The primers were synthesized by Sangon Biotech Co. Ltd. The expression level of SDF-1 was measured using RT-PCR, with GAPDH as the internal standard.

**Table 1:** Gene primer sequences used for RT-PCR

Primer	Forward	Reverse
SDF-1	5′-ATG CCC ATG CCG ATT CTT -3’	5’-GCA CAC TTG TCT GTT GTT GTT-3’
GAPDH	5’-CAG GAG GCA TTG CTG ATG AT-3’	5’-GAA GGC TGG GGC TCA TTT-3’

### Statistical analysis

Quantitative data were expressed as mean ± standard deviation. The data of each group were statistically analyzed using the SPSS Statistics 21.0 software. One-way analysis of variance was used to compare between groups. A *P* value < 0.05 indicated a statistically significant difference.

## Results

### Isolation and identification of human periodontal ligament stem cells

**Fig. 1:**
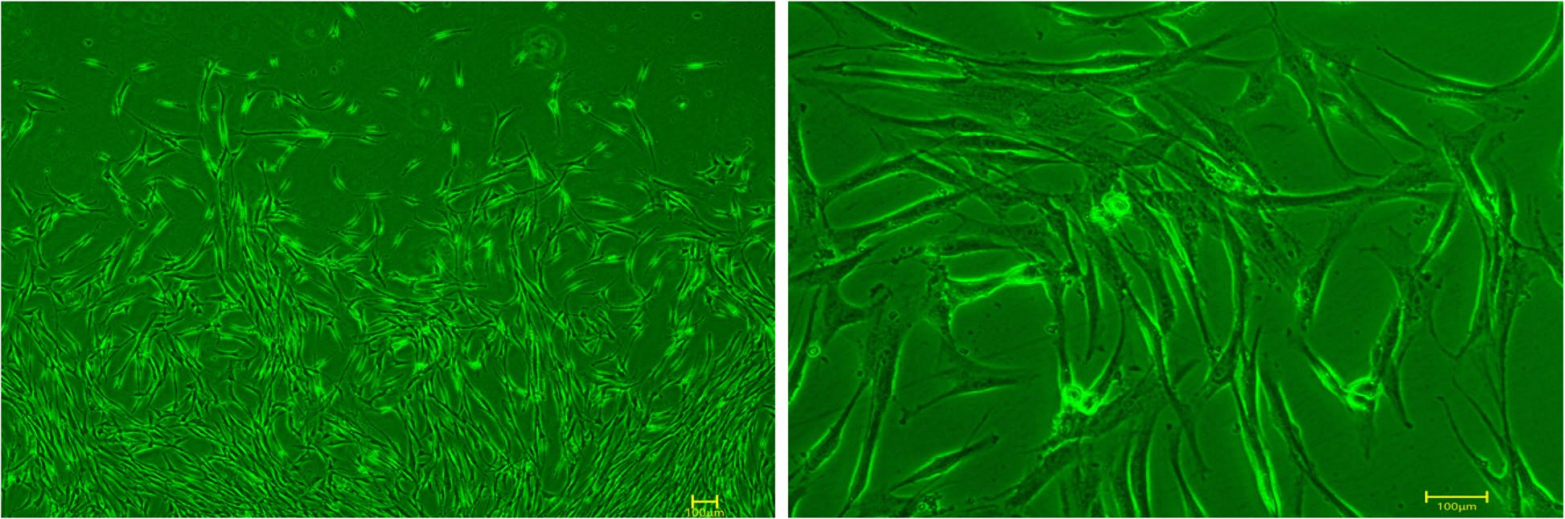
The primary culture cells of hPDLSCs

**Fig. 2:**
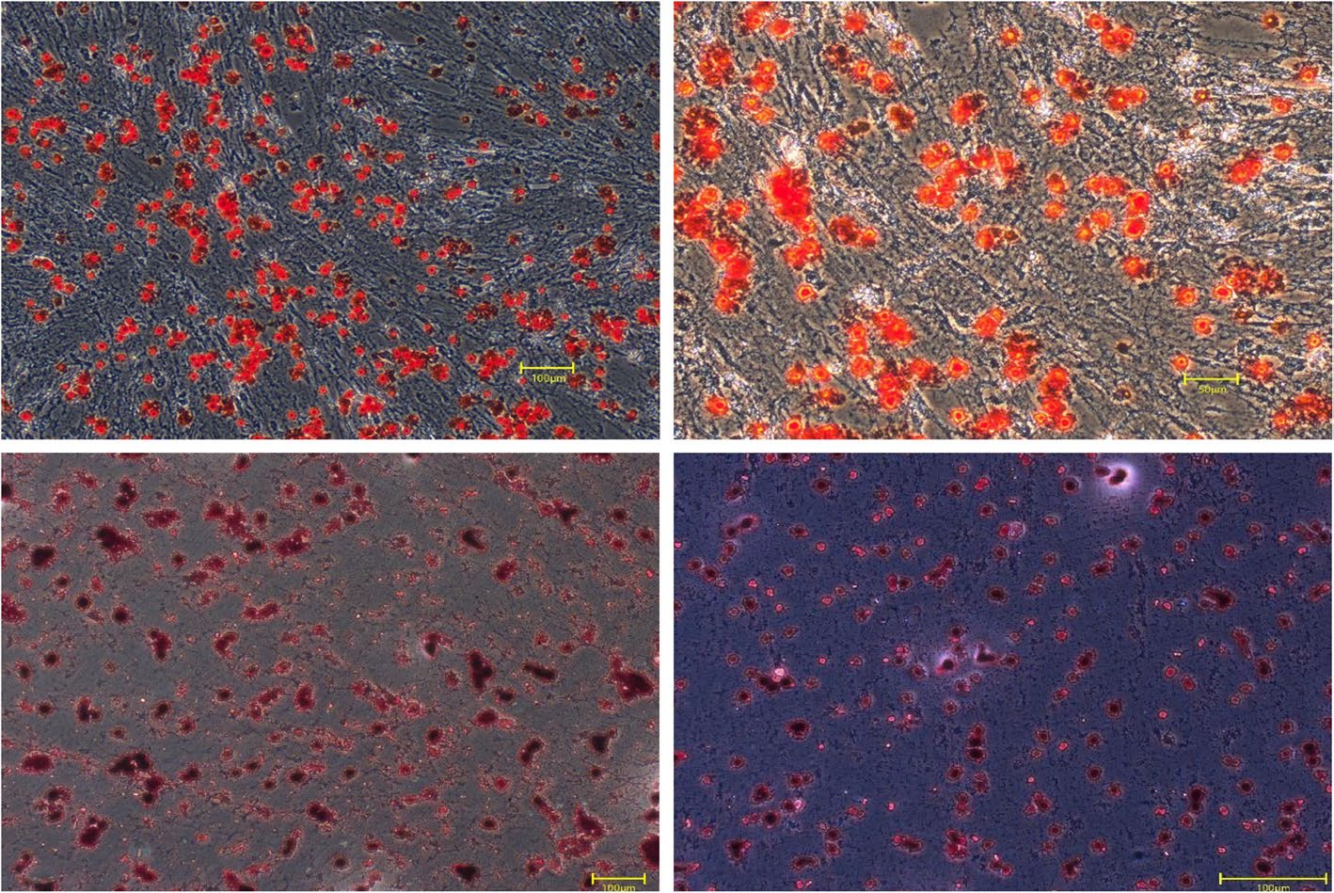
Alizarin red and Oil red O staining was positive

**Fig. 3:**
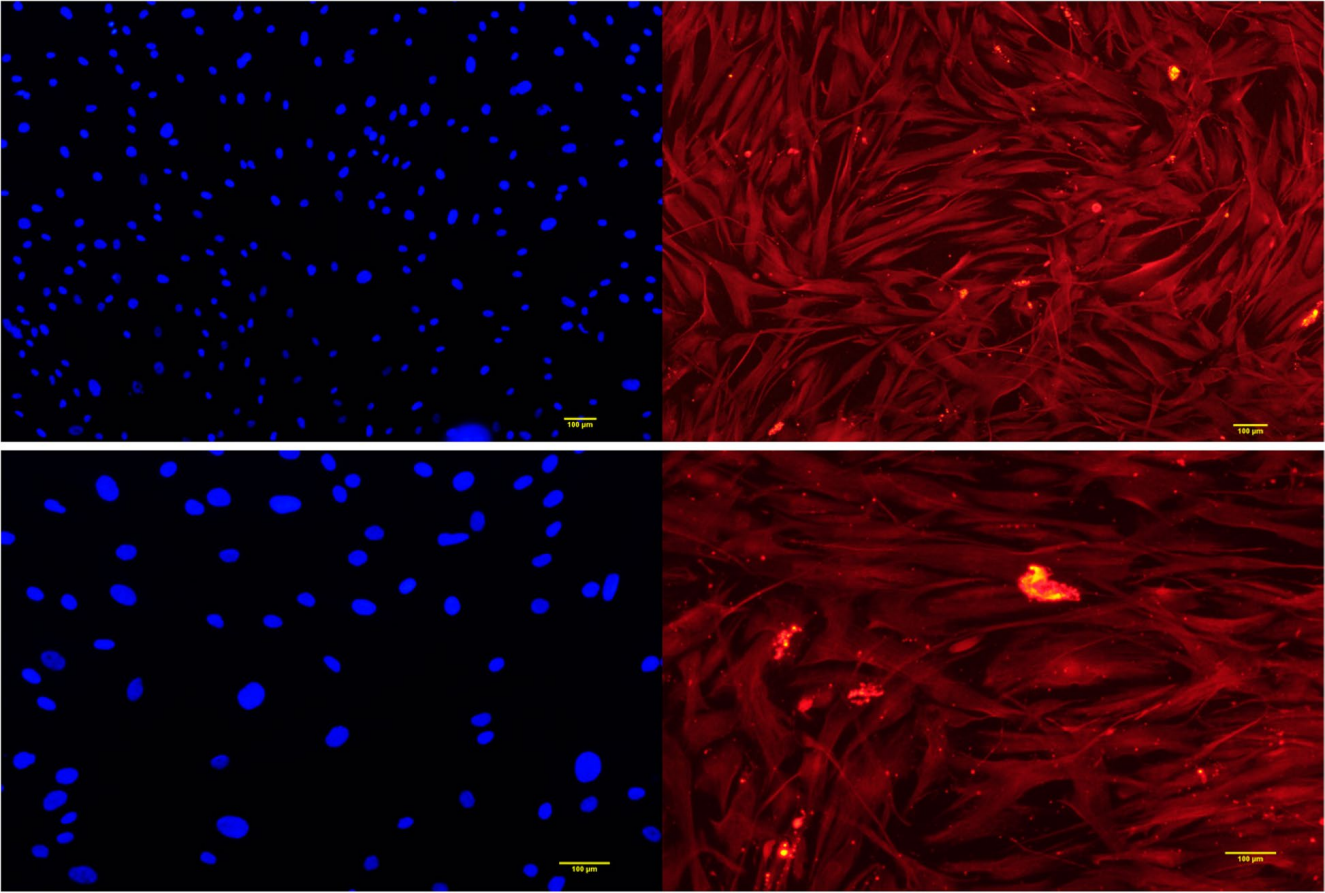
STRO-1 fluorescent staining was positive

**Fig. 4:**
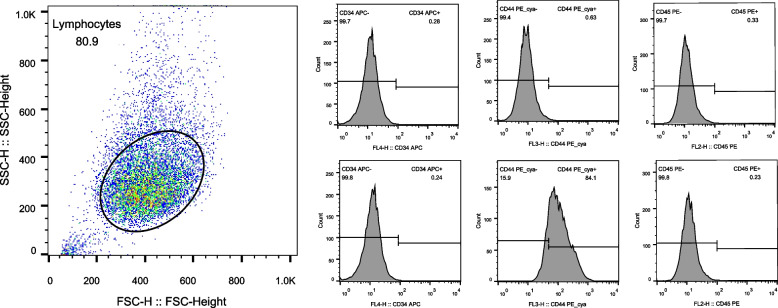
Flow cytometry detection results of hPDLCs

The h PDLSCs cultured by the tissue block method were observed under an inverted microscope. They were long, spindle-shaped monocytes with uniform cytoplasm and radial or vortex-like arrangement, as shown in Fig. 1. After 21 days of osteogenic and adipogenic induction, Alizarin red staining was positive and mineralized nodules were observed, as shown in Fig. 2. Oil red O staining was positive, and small fat droplets were observed, as shown in Fig. 2. Immunofluorescence showed that STRO-1 staining was positive (Fig. 3). The positive rate of cell surface marker CD44 was 84.1%, and the negative rate of hematopoietic markers CD34 and CD45 was 99.8% (Fig. 4). The results showed that the fibroblasts cultured by the tissue block method were h PDLSCs.

### CCK-8 proliferation test results

**Fig. 5:**
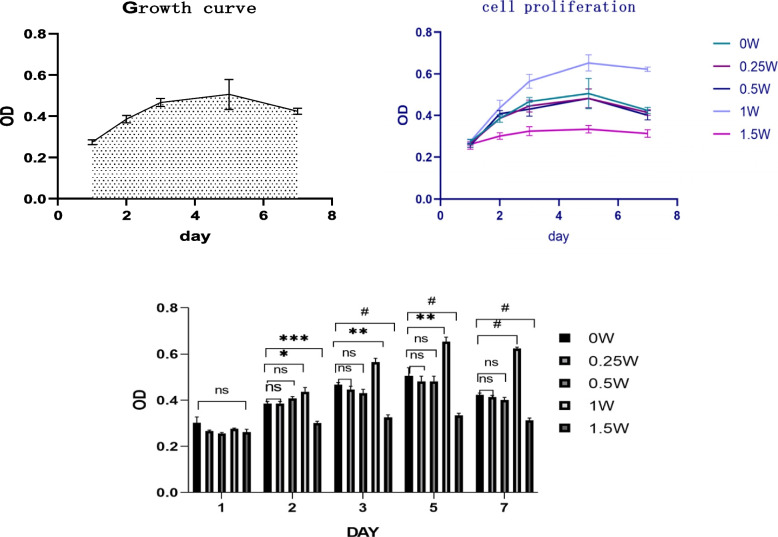
The cell growth curve of hPDLCs. ns represents *P* > 0.05,*represents *P* < 0.05, ** represents *P* < 0.01, *** represents *P* < 0.001, # represents *P* < 0.0001

On the second, third, fifth, and seventh days, when the Nd: YAG laser power was 1 W, the OD value increased (*P* < 0.05), and the proliferation accelerated. When the power was 1.5 W, the OD value decreased (*P* < 0.05) and the proliferation slowed down. When the power was 0.25 and 0.5 W, no significant difference was observed in OD values (*P* > 0.05) (Fig. 5).

### Transwell test

**Fig. 6:**
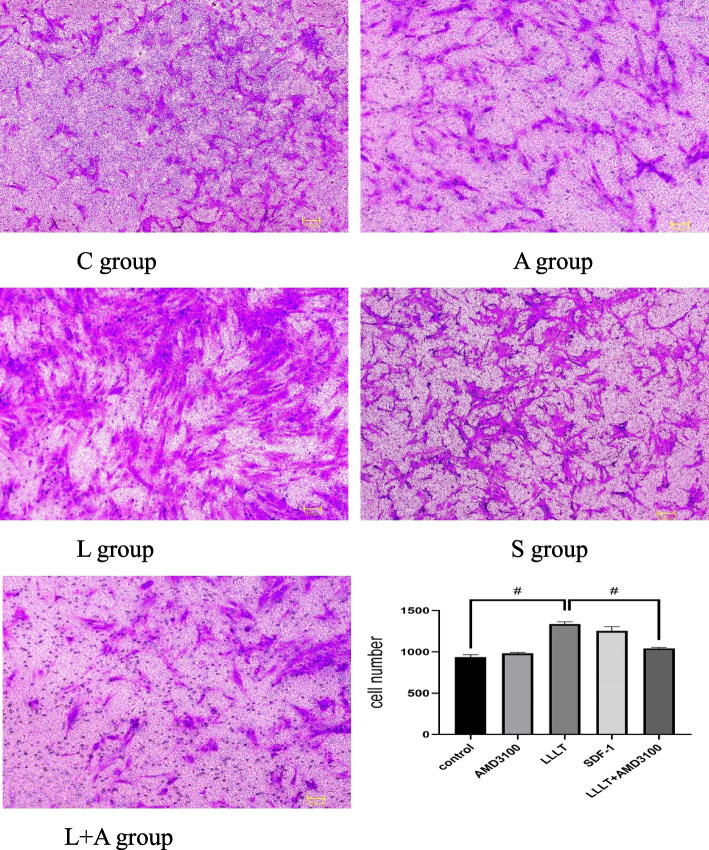
The Transwell test of hPDLCs(Inverted Microscope, × 10) # represents *P* < 0.0001

The number of cell perforations in different groups was as follows: C (956.5 ± 51.74), A (981.5 ± 21.15), S (1253 ± 87.21), and N (1336 ± 48.54). The number of cell perforations increased significantly after laser irradiation (*P* < 0.05). The number of cell perforations in the A and N + A groups (1044 ± 22.13) was significantly lower than that in the N group (*P* < 0.05). The statistical analysis showed that the number of cell perforations was higher in the N and S groups than in the C group, with significant differences (*P* < 0.05) (Fig. 6).

### ELISA and determining the mRNA expression level of SDF-1 genes

**Fig. 7:**
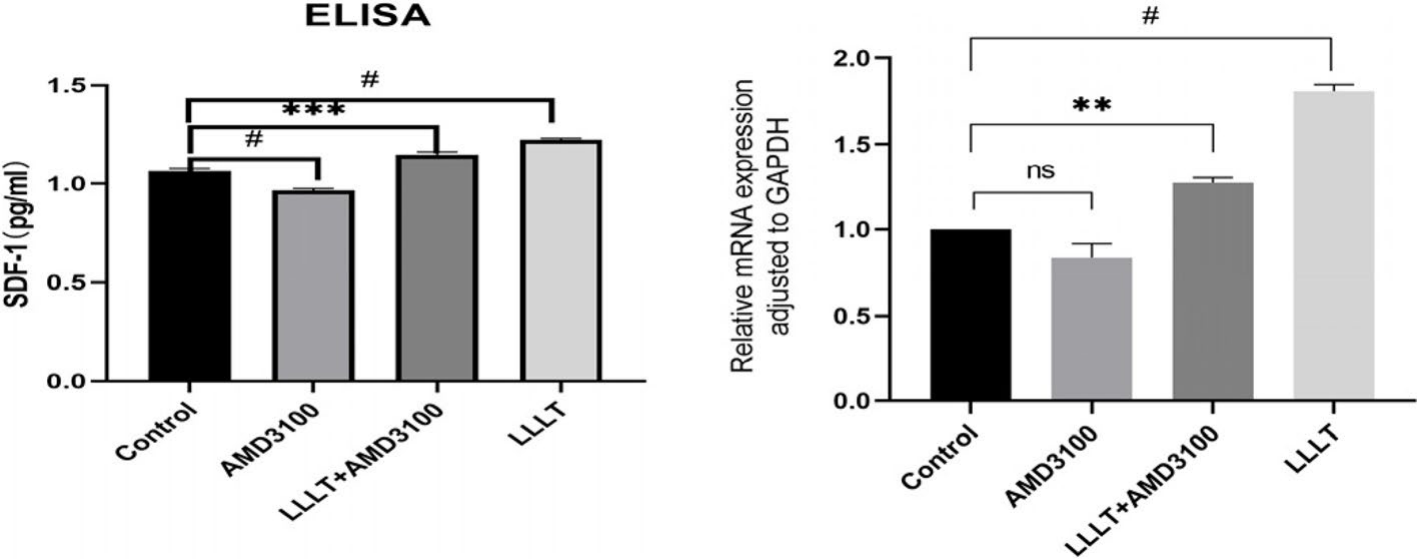
The ELISA test of hPDLCs. The RT-PCR test of hPDLCs. ns represents *P* > 0.05,*represents *P* < 0.05, ** represents *P* < 0.01,*** represents *P* < 0.001, # represents *P* < 0.0001

The level of SDF-1 and the relative expression of genes in the supernatant of cells increased in the Nd: YAG laser irradiation group compared with the control group (*P* < 0.05). The level of SDF-1 and the relative expression of genes decreased after adding the blocker AMD3100 (*P* < 0.05) (Fig. 7).

## Discussion

The endogenous regeneration of periodontal tissue can avoid complex treatment processes and reduce costs, which is a new concept of periodontal regeneration therapy [11]. Different optical parameters of low-energy lasers regulate cell function by stimulating or inhibiting responses [12, 13], and photobiomodulation therapy of low-energy lasers can optimize the environmental conditions of newly implanted cells. If the application of low-energy lasers can help improve the homing efficiency of periodontal-related stem cells, its social benefits and clinical application promotion value are extremely high. However, reports on this application research are few.

A variety of mesenchymal stem cells have multi-differentiation potential in the oral cavity. Mesenchymal stem cells are found in the dental pulp, dental sac, apical papilla, gingiva, and periodontal ligament [10]. Dental stem cells have a better regeneration effect on periodontal tissue than nondental stem cells [14]. Among the numerous dental mesenchymal stem cells, PDLSCs are easy to obtain and are the best candidate cells for periodontal regeneration, but they are prone to aging due to the influence of the number of passages of receptors [15]. Compared with the enzymatic digestion method, the tissue block method can obtain a more specific cell population, and the cultured cells have a higher proliferation rate and cell viability [16–18]. In 2006, the International Society for Cell Therapy proposed a set of markers and cell characteristics, mainly including self-renewal ability, multi-directional differentiation potential, expression of characteristic surface markers, and lack of expression of CD14, CD34, CD45, and human leukocyte antigen-DR (HLA-DR) [19–21]. In this experiment, hPDLSCs isolated by the tissue block method were spindle-shaped, similar to fibroblasts, and grew well. At the same time, they could differentiate into osteoblasts and adipocytes, indicating that they had the good self-renewal ability and multi-directional differentiation potential. Immunofluorescence experiments revealed specific expression of the early mesenchymal stem cell surface marker STRO-1. CD29 expression was positive, but the hematopoietic cell marker CD34 [22] and the surface marker CD45 of the whole white blood cell line were not expressed, which proved that the obtained cells were nonhematopoietic cell sources. It laid a solid foundation for subsequent experiments.

Photobiomodulation therapy is called PBM [23, 24], which promotes cell growth, regeneration, and tissue healing by emitting visible red to near-infrared light from coherent and incoherent light sources (low-energy laser, LED, and semiconductor laser). Studies have found that red light and near-infrared light can promote cell proliferation and differentiation; red light can increase cell viability; and yellow, orange, and green lights can promote cell migration [25]. The pulsed wave mode is more effective than the continuous wave mode in applying low-energy lasers to many biological systems [26]. This may be related to the delayed luminescence-enhanced alkaline phosphatase activity. The optical parameters and application dose of the low-energy lasers are the basis of laser photobiotherapy [27]. Semiconductor lasers with different wavelengths have a positive effect on the proliferation, migration, and osteogenic differentiation of periodontal ligament stem cells under different laser parameters. h PDLSCs were irradiated by 810 nm semiconductor laser with a energy density of 3.9 J/cm^2^ within 24–48 h, the proliferation accelerated, and MMP-8 protein expression decreased [28, 29]. However, the effect of an Nd: YAG laser on PDLSCs is rarely reported. An Nd: YAG laser is a deep-penetrating coherent pulsed infrared laser, and its photobiological regulation is commonly used in clinical practice. The clinical use of appropriate Nd: YAG laser parameters can improve the clinical effect of periodontal treatment [30, 31]. Therefore, an Nd: YAG laser was selected in this study.

Some scholars exposed stem cells to helium–nitrogen and semiconductor lasers with the energy density of 0.5–4 J/cm^2^ and power of 1–500 m W. This could increase the proliferation rate of multiple cell lines. The cell proliferation rate depended on the change in laser irradiation dose [32]. Some scholars also believed that the proliferation rate of cells was affected by the time of laser irradiation. They proposed that if the cells were given enough exposure at a low-energy level, the proliferation of cells and the secretion of macromolecules were regulated to a certain extent [33, 34]. In this study, the cells were divided into five groups based on the power of the Nd: YAG laser (1064 nm, MSP, 10 Hz, 30 s, 300 μ m): 0, 0.25, 0.5, 1, and 1.5 W. The CCK-8 cell proliferation assay showed that under the same conditions of other parameters, the proliferation rate of h PDLSCs was not significantly improved when the power of the Nd: YAG laser was 0.25 and 0.5 W. When the power was 1 W, the proliferation of h PDLSCs was significantly accelerated. When the power was 1.5 W, the proliferation rate of h PDLSCs did not increase but decreased, which might be related to Arndt–Schultz’s law; that is, the laser had a therapeutic window within a certain dose range. If the dose was too small, no effect was observed; if the dose exceeded this range, unnecessary inhibition occurred [35]. In this study, it was found that under the same other parameters, the power of the Nd: YAG laser between 0.5 W and 1.5 W irradiation might have an effect on the proliferation of h PDLSCs, and more than 1.5 W irradiation would have an inhibitory effect. It reflected the biphasic dose and wavelength effects at the low-energy level [36]. Therefore, the rational use of laser energy was extremely necessary. This was consistent with the effect of low-energy lasers using different treatment procedures on the proliferation and differentiation of dental pulp stem cells [37].

In most cases, mesenchymal stem cells are in a dormant state. When they receive signals regarding body damage, they can migrate to damaged tissues. The stromal-derived factor 1 (SDF-1) can recruit mesenchymal stem cells and other cells to participate in tissue repair and regeneration [38, 39], and can be used as a signal molecule to recruit and attract h PDLSC homing [40–42]. h PDLSCs are considered to be responsible for the homeostasis and regeneration of periodontal tissue. The h PDLSCs were cultured in DMEM with SDF-1, and the cell viability was analyzed using MTT and BrdU incorporation. It was confirmed that SDF-1 could improve the proliferation and survival of PDLSCs [43]; Jin et al. [44] showed that SDF-1 was an effective in vivo attractant acting on mesenchymal stem cells through the SDF-1/CXCR4 pathway. Zhang et al. [45] detected the mRNA and protein expression levels of CXCR4 and osteogenic factors (BMP-2, Runx2, and OPN) by establishing models in wild-type rats and SDF-1 gene knockout rats. The results showed that the upregulation of SDF-1 could promote the activation of the SDF-1/CXCR4 pathway. Studies using mass spectrometry analysis and Western blotting showed that SDF-1 played a leading role in SC-EVs, promoting pulp tissue regeneration [46]. SDF-1 is an important stem cell homing factor, and its signal transduction is first started by binding to its specific receptor CXCR4. CXCR4 is widely expressed in odontogenic stem cells [47, 48].

Transwell migration experiments showed that the migration ability of h PDLSCs was enhanced after Nd: YAG laser irradiation (1 W), and was stronger than that in the SDF-1 factor group. This showed that the Nd: YAG laser might have a similar effect as SDF-1. Therefore, the Nd: YAG laser might promote the migration of h PDLSCs by increasing the level of SDF-1 to affect the SDF-1/CXCR4 signaling pathway. AMD3100 is a potent chemical synthetic molecular inhibitor. Basheer et al. believed that AMD3100 could significantly inhibit the chemotactic effect of SDF-1 on bone marrow mesenchymal stem cells [49]. When AMD3100 was added in this experiment, the migration effect of h PDLSCs was weakened, probably because AMD3100 inhibited the function of CXCR4 as a chemotactic receptor [50, 51]. At the same time, the results of ELISA and PCR showed that the concentration and relative expression of SDF-1 were higher in the Nd: YAG laser treatment group than in the control group, while the concentration and relative expression of SDF-1 decreased after the addition of the blocker AMD3100. This suggested that the Nd: YAG laser might promote the homing of h PDLSCs through the SDF-1/CXCR4 signaling pathway at the protein and gene levels, which was one of the possible mechanisms of Nd: YAG laser-mediated periodontal endogenous regeneration. However, after adding the AMD3100 inhibitor, some h PDLSCs still migrated to the lower chamber, suggesting that the low-energy laser regulated the complexity of the h PDLSC homing mechanism through the SDF-1/CXCR4 signaling pathway. Studies found that SDF-1 regulated cell migration by activating multiple signaling pathways such as calcium release, MAPK p42/44 phosphorylation, and PI3K–Akt–NF-κ B activation [52]. Low-energy laser irradiation could activate Wnt/β-catenin protein, BMPs, and FGF signaling pathways related to the maintenance of stem cell types.

The mechanism of mesenchymal stem cell homing has not been elucidated to date, and homing is a key step to achieve the endogenous regeneration of periodontal tissue. Therefore, the strategy of optimizing stem cell homing is worthy of further exploration. However, the molecular mechanism of low-energy lasers triggering the homing of PDLSCs through the SDF-1/CXCR4 signaling pathway and promoting endogenous periodontal tissue regeneration remains to be further explored. This study was limited to the in vitro experimental stage. Therefore, the mechanism of Nd: YAG for periodontal endogenous regeneration needs to be further improved.

## Conclusions

A low-energy Nd: YAG laser may have a chemotactic effect similar to that of SDF-1. Nd: YAG laser irradiation with appropriate parameters can promote the proliferation and migration of h PDLSCs and improve the homing efficiency of mesenchymal stem cells. It provides a new method for the endogenous regeneration of periodontal tissue to improve homing efficiency. The SDF-1/CXCR4 signaling pathway may be involved in regulating the effect of LLLT on the homing of h PDLSCs, which is one of the possible mechanisms of LLLT-mediated periodontal regeneration.

## Data Availability

The datasets used and/or analysed during the current study available from the corresponding author on reasonable request.

## Abbreviations

SDF-1: Stromal cell-derived factor 1
CXCR4: Chemokine receptor
FBS: Fetal bovine serum
DMEM: Dulbecco’s Modified Eagle Medium
GAPDH: Glyceraldehyde-3-phosphate dehydrogenase
RT-PCR: Reverse transcription polymerase chain reaction
ELISA: Enzyme linked immunosorbent assay
SC-EVs: Extracellular vesicles
PFA: Paraformaldehyde
DAPI: 4′,6-Diamidino-2-phenylindole
hPDLSCs: Human periodontal ligament stem cells
Nd:YAG: Neodymium laser
